# Caregiver Burden and 30-Day Emergency Department Revisits

**DOI:** 10.1001/jamanetworkopen.2025.31166

**Published:** 2025-09-09

**Authors:** Nathalie Germain, Annie Toulouse-Fournier, Rawane Samb, Émilie Côté, Vanessa Couture, Stéphane Turcotte, Michèle Morin, Yves Couturier, Lucas B. Chartier, Nadia Sourial, Samir K. Sinha, Don Melady, Marie-Soleil Hardy, Richard Fleet, France Légaré, Denis A. Roy, Holly O. Witteman, Éric Mercier, Josée Rivard, Marie-Josée Sirois, Joanie Robitaille, Patrick M. Archambault

**Affiliations:** 1 Centre de recherche intégrée pour un système apprenant en santé et services sociaux, Centre intégré de santé et de services sociaux de Chaudière-Appalaches, Lévis, Québec, Canada; 2 Faculté de médecine, Université Laval, Québec, Québec, Canada; 3 VITAM–Centre de recherche en santé durable, Québec, Québec, Canada; 4 Department of Social Work, Université de Sherbrooke, Sherbrooke, Québec, Canada; 5 Department of Emergency Medicine, University Health Network, Toronto, Ontario, Canada; 6 Département de gestion, d’évaluation et de politique de santé, École de santé publique, Université de Montréal, Montreal, Québec, Canada; 7 Department of Family and Community Medicine, University of Toronto, Toronto, Ontario, Canada; 8 Department of Medicine, University of Toronto, Toronto, Ontario, Canada; 9 Department of Medicine, Sinai Health System and University Health Network, Toronto, Ontario, Canada; 10 Schwartz-Reisman Emergency Medicine Institute, Mount Sinai Hospital, Toronto, Ontario, Canada; 11 Faculty of Nursing, Université Laval, Québec, Québec, Canada; 12 Department of Family Medicine and Emergency Medicine, Université Laval, Québec, Québec, Canada; 13 Centre de recherche du CHU de Québec–Université Laval, Axe santé des populations et pratiques optimales en santé, Université Laval, Québec, Québec, Canada; 14 École des sciences de la readaptation, Faculté de médecine, Université Laval, Québec, Québec, Canada

## Abstract

**Importance:**

Caregivers of community-dwelling older adults play a protective role in emergency department (ED) care transitions. When the demands of caregiving result in caregiver burden, ED returns can ensue.

**Objective:**

To develop models describing whether caregiver burden is associated with ED revisits and hospital admissions up to 30 days after discharge from an initial ED visit.

**Design, Setting, and Participants:**

This prospective cohort study nested within the LEARNING WISDOM longitudinal cohort study included dyads of community-dwelling patients aged 65 years or older and their caregivers. Included patients were triaged to an observation unit stretcher on their index visit to 1 of 4 EDs within an integrated health multisite organization of 4 acute care hospitals in Québec, Canada, between January 1, 2019, and December 21, 2021, and underwent a transition of care when discharged back to the community. Analyses were conducted in May 2024.

**Exposure:**

Caregiver burden, collected using the brief 12-item Quebec French version of the Zarit Burden Interview (ZBI; score range, 0-48, with higher scores indicating higher burden).

**Main Outcomes and Measures:**

Revisits to the ED, defined as a return to any ED in the 4-hospital network, within 3, 7, or 30 days of the index visit and return visits to the ED resulting in hospitalization within 30 days of the index visit. Moderation of outcomes by wave of the COVID-19 pandemic at the index visit was also assessed.

**Results:**

Among 1409 caregiver-patient dyads, 711 patients (50.5%) and 980 caregivers (69.6%) were women; mean (SD) age was 77.06 (7.39) years for patients and 63.87 (12.04) years for caregivers. The mean (SD) ZBI score was 7.33 (7.11). Caregivers were most often spouses of patients (667 [48.0%]) or children of patients (534 [37.9%]). Among all patients, 75 (5.3%) returned to the ED within 3 days, 133 (9.4%) returned within 7 days, 292 (20.7%) returned within 30 days, and 88 (6.2%) were admitted to the hospital within 30 days. Each point increase on the ZBI scale was associated with an increase in the odds of a 30-day revisit to the ED (odds ratio [OR], 1.03; 95% CI, 1.00-1.05; *P* = .03), but associations were not found in models with shorter time windows (3 days: OR, 1.01; 95% CI, 0.98-1.04; *P* = .69; 7 days: OR, 1.01; 95% CI, 0.98-1.03; *P* = .55) or for revisits with hospital admissions (OR, 1.02; 95% CI, 0.99-1.05; *P* = .24). Associations between ZBI scores and 30-day ED revisits may have been moderated by the COVID-19 pandemic waves: the first interwave period (between waves 1 and 2) reversed the association (OR, 0.89; 95% CI, 0.78-0.97).

**Conclusions and Relevance:**

The findings suggest caregiver burden may be associated with ED revisits within 30 days of discharge from an initial ED visit among community-dwelling older adults. Future studies could enhance the management of ED revisits by demonstrating the longitudinal impact of caregiver burden on ED use in older adults.

## Key Points

**Question:**

Is caregiver burden among caregivers of community-dwelling older adult patients associated with emergency department (ED) revisits and revisits resulting in hospitalization within 30 days of discharge from an initial ED visit?

**Findings:**

In this cohort study of 1409 dyads of patients aged 65 years or older and their caregivers, higher caregiver burden was associated with a modest increase in the likelihood of an ED revisit within 30 days, though not with shorter-term revisits or revisits with hospitalizations.

**Meaning:**

The findings suggest reducing caregiver burden might help prevent returns to the ED within 30 days among community-dwelling older adults.

## Teaser

This cohort study explores whether higher caregiver burden among caregivers of community-dwelling older adult patients is associated with emergency department revisits with or without hospitalization within 30 days of discharge from an initial visit.

## Introduction

Older adults are frequent users of emergency department (ED) services.^1,2^ ED return visits after discharge from an index ED visit by older adult patients is a substantial factor in ED overuse.^3^ Frequent ED use for complex needs in this population is considered suboptimal and may indicate that those needs have not been adequately addressed.^4,5,6,7^ Older adults are vulnerable to adverse outcomes related to ED visits, due in part to poor care transitions from the ED to community, declines in functional autonomy, lack of social support once discharged from the ED, comorbidities, and polypharmacy.^8^ Both ED use and resource use intensity (eg, diagnostic testing, consultation, or length of stay) appear to increase with age.^8^ Informal or family caregivers are often called upon to support care transitions.^9,10^

Caregivers protect the health of those in their care^11^ and are also often included in assessments and decision-making.^12^ As part of their role in patient care, they may endure physical, emotional, social, and financial strain, known collectively as caregiver burden.^13,14^ Caregiver burden is associated with higher patient mortality^15^ and with hospitalization.^16^ Higher caregiver burden is associated with greater ED use.^17^ Driving factors may include dependency on the caregiver,^18^ responsive behavior in care recipients,^19^ and unmet supportive care needs.^20^ However, there is a knowledge gap in understanding if caregiver burden, operationalized with questionnaires, is associated with increased ED revisits in community-dwelling older adults shortly after discharge from the ED. Our objective was to describe whether caregiver burden is associated with unplanned ED revisits up to 30 days after discharge from an initial visit.

## Methods

### Study Design and Context

This prospective cohort study was nested within the LEARNING WISDOM longitudinal cohort study.^17^ We report our findings in accordance with the Transparent Reporting of a Multivariable Prediction Model for Individual Prognosis or Diagnosis (TRIPOD)^21^ and Strengthening the Reporting of Observational Studies in Epidemiology (STROBE)^22^ reporting guidelines. The protocol for this study was approved by the ethics review committee of the Centre intégré de santé et de services sociaux de Chaudière-Appalaches (CISSS-CA) in Québec, Canada. Participants provided oral informed consent. The LEARNING WISDOM cohort included older adults who underwent a transition of care following a visit to 1 of the 4 EDs within the CISSS-CA between January 1, 2019, and December 21, 2021, and their caregivers. The CISSS-CA is an integrated health organization consisting of 4 acute care hospitals: Hôtel-Dieu de Lévis, Hôpital de Saint-Georges, Hôpital de Montmagny, and Hôpital de Thetford Mines.

### Participants

The LEARNING WISDOM cohort included consenting patients aged at least 65 years who had been discharged back to the community from the ED after being triaged to an observation unit stretcher on their index visit. Patients only seen in the ambulatory care section of the ED, admitted to the hospital, transferred to another hospital, or transferred to a long-term care center following the index visit were excluded. Caregivers of older patients were informal noncompensated caregivers, usually family members or friends, who provided support and assistance to patients in the cohort. Patients and their caregivers were required to understand French.

### Data Collection

As part of a continuous quality improvement project led by the CISSS-CA, patients were contacted by telephone between 24 hours and up to 7 days after ED discharge.^23^ Patients were then invited to participate in a more in-depth research interview in the following days, and during this second call, both patients and their caregivers were required to summarize—in their own words—their understanding of the study, based on the Nova Scotia Criteria to demonstrate informed consent.^24^ After patients participated, they were asked if they consented to have their caregivers contacted by the research team. We conducted a structured interview to obtain demographic characteristics, followed by administering the Québec French version of the 12-item brief Zarit Burden Interview (ZBI) to all participating caregivers.^25^

### Measures

We extracted hospital administrative data from the Med-GPS and Med-Urge (Logibec) databases. Demographic and questionnaire data were collected using REDCap (Vanderbilt University) by trained research professionals.^26,27^

The ZBI is the most widely used instrument measuring caregiver burden, with internal consistency indices ranging between 0.7 and 0.9.^28,29,30^ In the 12-item ZBI, questions include items about strain in the caregiver’s role and personal life associated with caregiving. Each question is scored by frequency on a 5-point Likert scale (0 to 4, with 0 indicating “never”; 1, “rarely”; 2, “sometimes”; 3, “fairly often”; and 4, “always”), and scores are summed, with higher scores indicating a higher degree of burden (total score range, 0-48).

Outcome variables included patient revisits to the ED and hospitalizations on revisit. Revisits to the ED were defined as whether a given patient returned to any ED in the 4-hospital network within 3, 7, or 30 days of the index visit for any reason. Hospitalizations included returns to the ED occurring within 30 days after the index visit that resulted in hospitalization. Index visits were defined as the patient’s first visit to the ED that required triage to a stretcher in the observation unit. Revisit intervals are associated with different outcomes; early revisits within 3 to 7 days are generally considered failures of care coordination, while at 30 days, they are considered to be due to multifactorial factors of the care transition.^31,32,33,34,35^

Covariates included both patient and caregiver characteristics collected by trained research personnel over the telephone. For patients, we collected age, sex, income, educational level, living situation (home with family, home alone, or living in a care or retirement home), access to a family doctor, access to an appointment with a family doctor in a reasonable time frame, access to transport, and precedent visits to the ED over the past year. We also collected patient comorbidities using the Charlson Comorbidity Index (CCI),^36^ whether patients arrived by their own means or arrived by ambulance, the Canadian Triage and Acuity Scale (CTAS) level (ranging from 1 [requiring immediate care] to 5 [requiring nonurgent care]),^37^ and the time spent on a stretcher at the ED. For the CCI, we removed points allocated according to the age of the patient to consider age as an independent predictor variable. Caregiver characteristics of interest included age, sex, ethnicity, income, educational level, housing, and the nature of the caregiver–care recipient relationship (spouse, child-parent, or other).

We also included the wave of COVID-19 pandemic at the time of the index visit according to Québec public health authorities. Data aggregation was performed to maximize the distinguishability of each stratum. Data grouping decisions can be found in eAppendix 1 in Supplement 1.

### Statistical Analysis

We performed a priori analyses to determine the estimated power to detect outcomes of interest (eAppendix 2 in Supplement 1). In simulations using normally distributed ZBI scores, 700 patients were sufficient to achieve statistical power (80%), and we estimated that models could accommodate a maximum of 3 covariates and 3 interaction terms, with ZBI scores as the predictor variable.^38,39^

Using logistic regression modeling with a purposeful selection algorithm^40,41^ (eAppendix 3 in Supplement 1), we analyzed whether caregiver burden among caregivers of older adult patients was associated with ED revisits and ED revisits resulting in hospitalization.^42,43^ All available clinically relevant data (eAppendix 1 in Supplement 1) were used in the development of the models. We also analyzed whether the COVID-19 pandemic period moderated the associations between caregiver burden, revisits, and hospital admissions. Data cleaning and analyses were conducted in R, version 4.3.0 (R Project for Statistical Computing). All tests were 2-sided, with a significance threshold of *P* < .05. We ran the Hosmer-Lemeshow goodness of fit test.^40^ The test divides data into 10 groups based on predicted probabilities and compares the predicted and actual event counts in each group. A low *P* value indicates poor model fit.

ZBI scores may have been biased by the timing of measurement. In some cases, due to delays in data collection, caregivers may have responded to the ZBI after the revisit to the ED occurred. As sensitivity and exploratory analyses, we tested whether the coefficients, significance levels, and model fit statistics differed between patient-caregiver dyads whose ZBI scores were collected before vs after the revisit occurred.

## Results

### Participants

**Figure 1.**
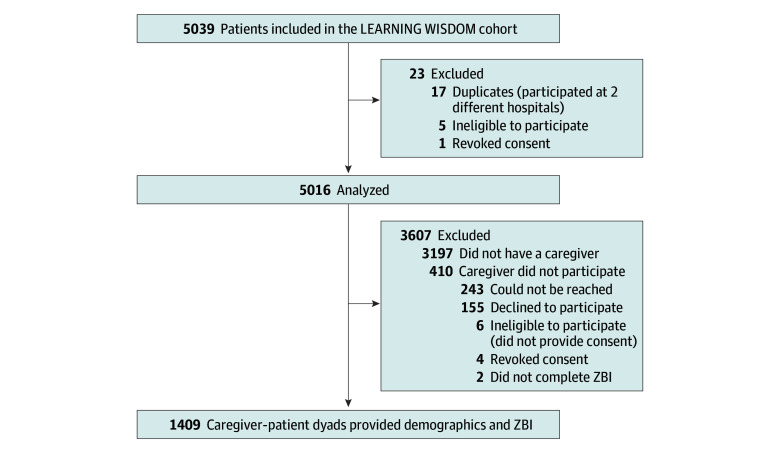
Flowchart Describing Recruitment of Patients and Their Caregivers ZBI indicates 12-Item Zarit Burden Interview.

The total LEARNING WISDOM cohort included 5016 participants of the larger study (Figure 1). Among these participants, 1819 allowed the research team to contact their caregiver, and 410 caregivers were excluded (6 were unable to provide informed consent, 161 declined to participate or withdrew their participation, and 243 could not be reached), leaving 1409 patient-caregiver dyads; 292 patients (20.7%) had an ED revisit with 30 days and 1117 (79.3%) did not. The patient sample analyzed in this substudy was comparable to the broader LEARNING WISDOM cohort in terms of age, sex, educational level, social support, access to transport, and ethnicity but demonstrated a higher burden of illness and a higher proportion of patients living alone, although the mean number of people in a patient’s social circle was similar in both groups. This study’s sample indicated less access to primary care or ability to get a physician’s appointment when needed compared with the broader cohort.

Among patients in the current study, 698 (49.5%) were men and 711 (50.5%) were women, and mean (SD) age was 77.06 (7.39) years. Among caregivers, women constituted the majority at 980 (69.6%), contrasting with men (429 [30.4%]), and mean (SD) age was 63.87 (12.04) years. Regarding caregiver-patient relationships, the largest proportion consisted of spouses at 667 (48.0%), followed by child-parent relationships at 534 (37.9%) and other family members or friends at 198 (14.1%). Among all patients, 292 (20.7%) returned to the ED within 30 days of the index visit, 133 (9.4%) revisited within 1 week, 75 (5.3%) revisited within 72 hours, and 88 (6.2%) experienced a revisit resulting in hospitalization within 30 days. The mean (SD) ZBI score was 7.33 (7.11). The internal consistency of ZBI scores was high (Cronbach α, 0.87; 95% CI, 0.86-0.89). Demographic characteristics stratified by patients who revisited the ED within 30 days are reported in Table 1.

**Table 1.** Demographic Characteristics of Patients and Their Caregivers

Characteristic	Patient-caregiver dyads (N = 1409)^a^	
	ED revisit within 30 d (n = 292)	No ED revisit within 30 d (n = 1117)
Patient sex		
Men	151 (51.7)	547 (49.0)
Women	141 (48.3)	570 (51.0)
Patient age, y		
Mean (SD)	76.7 (7.29)	77.2 (7.42)
Median (range)	76.0 (65.0-102.0)	76.0 (65.0-100.0)
Arrival method on index visit		
Ambulance	146 (50.0)	604 (54.1)
Ambulant	146 (50.0)	513 (45.9)
CTAS triage score on index visit^b^		
5	34 (11.6)	148 (13.2)
4	132 (45.2)	541 (48.4)
3	109 (37.3)	386 (34.6)
2	17 (5.8)	42 (3.8)
Hospital visited at index visit		
Saint-Georges	60 (20.5)	227 (20.3)
Hôtel-Dieu de Lévis	98 (33.6)	449 (40.2)
Montmagny	56 (19.2)	203 (18.2)
Thetford Mines	78 (26.7)	238 (21.3)
Time on stretcher at index visit, h		
Mean (SD)	11.12 (8.71)	11.57 (8.60)
Median (range)	8.39 (0.11-52.42)	8.72 (0.00-70.17)
Triage delay at index visit, h		
Mean (SD)	0.07 (0.16)	0.06 (0.15)
Median (range)	0 (0.00-1.20)	0 (0.00-1.32)
Visits to ED in past year, No.		
Mean (SD)	1.71 (2.19)	1.16 (1.62)
Median (range)	1.00 (0-16.00)	1.00 (0-12.00)
Social support persons, No.^c^		
Mean (SD)	4.05 (4.41)	4.11 (5.57)
Median (range)	3.00 (0-60.00)	3.00 (0-150.00)
COVID-19 period when index ED visit occurred^d^		
Prepandemic	114 (39.0)	484 (43.3)
Wave 1	41 (14.0)	158 (14.1)
Between waves 1 and 2	16 (5.5)	60 (5.4)
Wave 2	76 (26.0)	244 (21.8)
Wave 3	23 (7.9)	96 (8.6)
Wave 4	22 (7.5)	75 (6.7)
Charlson Comorbidity Index^36^		
Mean (SD)	5.61 (2.37)	5.35 (2.23)
Median (range)	5.00 (2.00-14.0)	5.00 (2.00-14.0)
Caregiver sex		
Men	91 (31.2)	338 (30.3)
Women	201 (68.8)	779 (69.7)
Caregiver age, y		
Mean (SD)	63.3 (12.0)	64.0 (12.0)
Median (range)	65.0 (28.0-89.0)	66.0 (24.0-92.0)
Patient has a family physician		
No	16 (5.5)	81 (7.3)
Yes	276 (94.5)	1036 (92.7)
Patient can quickly consult their family physician		
No	138 (47.3)	477 (42.7)
Yes	154 (52.7)	640 (57.3)
Patient has access to transport for medical care		
No	18 (6.2)	52 (4.7)
Yes	274 (93.8)	1065 (95.3)
Educational level		
Patient		
Primary school	141 (48.3)	534 (47.8)
Secondary school	116 (39.7)	464 (41.5)
University	35 (12.0)	119 (10.7)
Caregiver		
Primary school	68 (23.3)	212 (19.0)
Secondary school	183 (62.7)	736 (65.9)
University	41 (14.0)	169 (15.1)
Caregiver–patient relationship		
Other family or friend	42 (14.4)	156 (14.0)
Child–parent	116 (39.7)	418 (37.4)
Spouse	134 (45.9)	543 (48.6)
Annual income, CAD$		
Patient		
No response	70 (24.0)	305 (27.3)
<30 000	128 (43.8)	438 (39.2)
≥30 000	94 (32.2)	374 (33.5)
Caregiver		
No response	70 (24.0)	308 (27.6)
<50 000	130 (44.5)	467 (41.8)
≥50 000	92 (31.5)	342 (27.6)
Residence type		
Patient		
Care home	48 (16.4)	174 (15.6)
Home	179 (61.3)	725 (64.9)
Home alone	65 (22.3)	218 (19.5)
Caregiver		
Care home	7 (2.4)	17 (1.5)
Home	239 (81.8)	952 (85.2)
Home alone	46 (15.8)	148 (13.2)
ZBI score^e^		
Mean (SD)	8.55 (7.42)	7.01 (6.99)
Median (range)	7.00 (0-40.0)	5.00 (0-44.0)

Abbreviations: CAD, Canadian dollars; CTAS, Canadian Triage and Acuity Scale; ED, emergency department; ZBI, 12-Item Zarit Burden Interview.

^a^ Data are presented as number (percentage) of patient-caregiver dyads unless otherwise indicated.

^b^ CTAS of 1 requires immediate care, 2 requires emergent care and rapid medical intervention, 3 requires urgent care, 4 requires less-urgent care, and 5 requires nonurgent care.

^c^ Self-reported persons in social circle.

^d^ Waves correspond to the Institut national de santé publique du Québec definitions of the COVID-19 timeline in Québec: prepandemic (January 1, 2019, to March 12, 2020) and throughout waves 1 (March 13 to July 11, 2020), 2 (August 23, 2020, to March 20, 2021), 3 (March 21 to July 17, 2021), and 4 (July 18 to December 4, 2021).

^e^ Score range, 0-48, with higher scores indicating higher burden.

### Model of 30-Day Revisits

**Figure 2.**
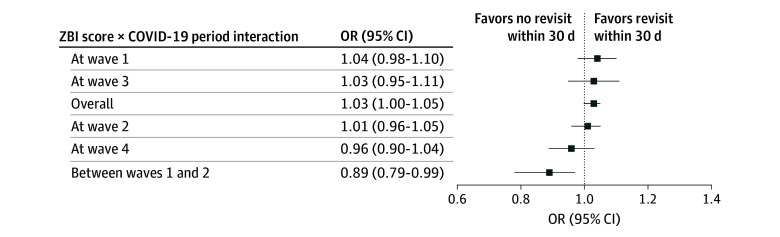
Moderation of Associations of 12-Item Zarit Burden Interview (ZBI) Scores and COVID-19 Periods With Odds of 30-Day Emergency Department Revisit COVID-19 waves correspond to the Institut national de santé publique du Québec definitions of the COVID-19 timeline in Québec: wave 1, March 13 to July 11, 2020; wave 2, August 23, 2020, to March 20, 2021; wave 3, March 21 to July 17, 2021; wave 4, July 18, 2021, to December 4, 2021. OR indicates odds ratio.

**Figure 3.**
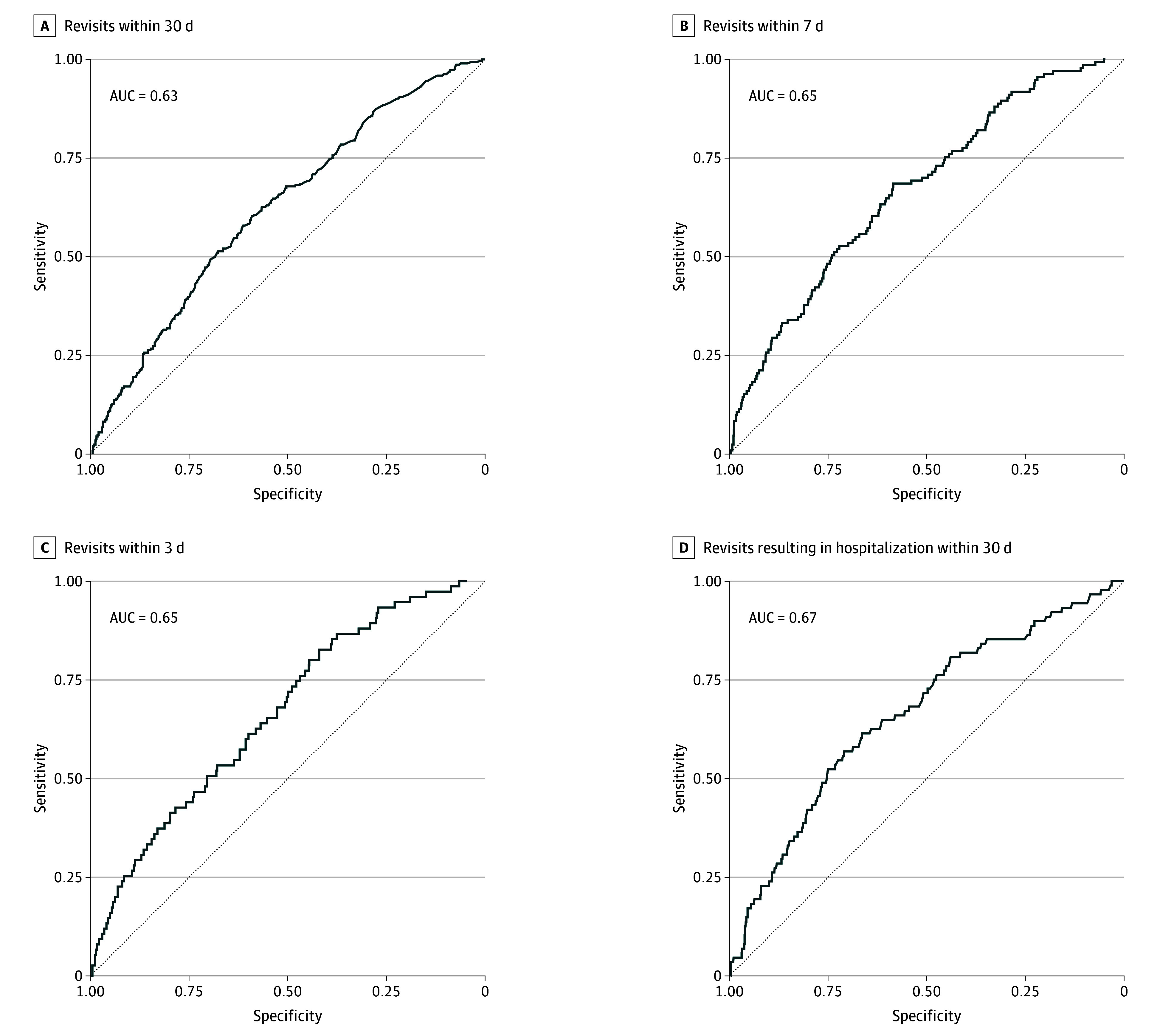
Receiver Operating Characteristic Curves for Each Logistic Regression Model A, Factors included 12-Item Zarit Burden Interview (ZBI) score, number of emergency department (ED) visits in the previous year, and the COVID-19 period (which interacted with both ZBI score and past-year ED visits). B, Factors included number of ED visits in the previous year, male patient sex, caregiver living in a care home, and Canadian Triage and Acuity Scale (CTAS) triage level below 5 on a scale of 1 to 5 (CTAS 1 requires immediate care and CTAS 5 requires nonurgent care). C, Factors included number of ED visits in the previous year, caregiver living in a care home, a CTAS triage score below 5, and time on a stretcher at the index ED visit. D, Factors included walk-in arrival at the index ED visit, Charlson Comorbidity Index score, annual caregiver income.

The logistic regression model that best explained 30-day revisits to the ED included ZBI scores, ED visits in the preceding year, and the COVID-19 period. The COVID-19 period did not have a statistically significant association with 30-day ED revisits or revisits with hospitalization but did appear to moderate associations of these revisits with both ZBI scores and ED visits in the past year (Table 2). Each added point on the ZBI scale was associated with an increase in the odds of an ED revisit within 30 days (OR, 1.03; 95% CI, 1.00-1.05; *P* = .03) while controlling for covariates. Similarly, each ED visit in the past year was associated with an increase in the odds of an ED revisit within 30 days (OR, 1.12; 95% CI, 1.01-1.24; *P* = .03). However, the first COVID-19 pandemic interwave appeared to reverse the association between ZBI scores and 30-day revisits (OR, 0.89; 95% CI, 0.79-0.99) (Figure 2). The model was a good fit of the data (goodness of fit [GOF] test, χ^2^ = 9.11; *P* = .33). The *C* statistic, representing the model’s discriminative capabilities, was low (0.63; SE, 0.02) (Figure 3).

**Table 2.** Logistic Regression Models for Odds of ED Revisits Within 30 Days^a^

Model	OR (95% CI)		*P* value^b^
	Univariate model	Multivariate model	
Main			
Per point increase in ZBI score	1.03 (1.01-1.05)	1.03 (1.00-1.05)	.03
Per ED visit in past year	1.17 (1.09-1.24)	1.12 (1.01-1.24)	.03
COVID-19 period			
Prepandemic	1 [Reference]	1 [Reference]	NA
Wave 1	1.10 (0.74-1.64)	0.61 (0.29-1.26)	.18
Between waves 1 and 2	1.13 (0.63-2.04)	2.27 (0.92-5.61)	.07
Wave 2	1.32 (0.95-1.84)	1.34 (0.78-2.31)	.28
Wave 3	1.02 (0.62-1.67)	0.63 (0.25-1.62)	.34
Wave 4	1.25 (0.74-2.09)	1.56 (0.69-3.51)	.29
Moderation of ZBI score by COVID-19 period			
Wave 1	NA	1.04 (0.98-1.10)	.17
Between waves 1 and 2	NA	0.89 (0.79-0.99)	.03
Wave 2	NA	1.01 (0.96-1.05)	.76
Wave 3	NA	1.03 (0.95-1.11)	.47
Wave 4	NA	0.96 (0.90-1.04)	.33
Moderation of past-year ED visits by COVID-19 period			
Wave 1	NA	1.23 (0.99-1.54)	.06
Between waves 1 and 2	NA	1.14 (0.81-1.59)	.45
Wave 2	NA	0.97 (0.81-1.15)	.70
Wave 3	NA	1.20 (0.91-1.58)	.20
Wave 4	NA	1.02 (0.80-1.29)	.89

Abbreviations: ED, emergency department; NA, not applicable; OR, odds ratio; ZBI, 12-item Zarit Burden Interview.

^a^ Waves correspond to the Institut national de santé publique du Québec definitions of the COVID-19 timeline in Québec: prepandemic (January 1, 2019, to March 12, 2020) and throughout waves 1 (March 13 to July 11, 2020), 2 (August 23, 2020, to March 20, 2021), 3 (March 21 to July 17, 2021), and 4 (July 18 to December 4, 2021).

^b^ For multivariate model.

### Model of 7-Day Revisits

Male sex (OR, 1.67; 95% CI, 1.13-2.47), ED visits in the previous year (OR, 1.15; 95% CI, 1.05-1.25), a CTAS triage level of 2 at the index visit (OR, 3.95; 95% CI, 1.54-10.18), and having a caregiver residing in a care home (OR, 3.84; 95% CI, 1.32-11.15) were positively associated with revisits within 7 days (eAppendix 4 in Supplement 1). ZBI scores were not statistically significantly associated with the probability of a 7-day ED revisit (OR, 1.01; 95% CI, 0.98-1.03; *P* = .55). The independent variables of patient living alone and ED length of stay on a stretcher were not statistically significant predictors but were confounders that contributed to model fit (eAppendix 4 in Supplement 1). There were no statistically significant interactions between the variables of sex, ED visits in the previous year, CTAS triage level, patient living situation, caregiver living situation, or time on stretcher at the ED; the ZBI; or the COVID-19 pandemic waves. The model was a good fit of the data (GOF, χ^2^ = 4.33; *P* = .83). The *C* statistic was low (0.65; SE, 0.02) (Figure 3).

### Model of 3-Day Revisits

ED visits in the past year (OR, 1.13; 95% CI, 1.02-1.26), a caregiver living in a care home (OR, 3.37; 95% CI, 1.10-10.33), and a CTAS triage level of 2 (OR, 7.14; 95% CI, 1.71-29.72), 3 (OR, 3.78; 95% CI, 1.13-12.61), or 4 (OR, 3.59; 95% CI, 1.09-11.86) were positively associated with revisits within 72 hours of the index visit (eAppendix 4 in Supplement 1). Only ED length of stay on a stretcher at the index visit (OR, 0.97; 95% CI, 0.94-1.00) was associated with lower odds of an ED revisit within 72 hours. ZBI scores were not significantly associated with 72-hour revisits (OR, 1.01; 95% CI, 0.98-1.04; *P* = .69). There were no statistically significant interactions between the variables of ED visits in the previous year, CTAS triage level, time on stretcher at the ED, or caregiver living situation; the ZBI; or the COVID-19 pandemic waves. The model was a good fit of the data (GOF, χ^2^ = 2.93; *P* = .94). The *C* statistic was low (0.65; SE, 0.03) (Figure 3).

### Model of 30-Day Hospitalizations

Several factors were significantly associated with an ED revisit within 30 days that resulted in hospitalization (eAppendix 4 in Supplement 1). These included arriving as a walk-in at the index visit (OR, 1.57; 95% CI, 1.01-2.43) and a higher CCI score (OR, 1.17; 95% CI, 1.07-1.29). Furthermore, compared with not reporting an income, reported annual caregiver income had a significant association with an ED revisit within 30 days, both for incomes of CAD$50 000 or more (OR, 2.12; 95% CI, 1.05-4.26) and for incomes less than CAD$50 000 (OR, 2.81; 95% CI, 1.47-5.38). ZBI scores were not significantly associated with 30-day revisits resulting in hospitalization (OR, 1.02; 95% CI, 0.99-1.05; *P* = .24). There were no significant interactions between the CCI, arrival method on index visit, caregiver annual income, and the ZBI. The model was a good fit of the data (GOF, χ^2^ = 8.49; *P* = .39). The *C* statistic was low (0.67; SE, 0.03) (Figure 3).

### Sensitivity Analyses

Most caregivers had their data collected before the patient revisited the ED (n = 1099 [78.0%]). However, 310 patients (22.0%) revisited the ED before their caregiver was recruited. To assess whether this moderated the association between ZBI scores and ED revisits within 30 days, we split the dataset into 2 groups (caregiver burden measured before ED revisit and after ED revisit) and reperformed the first model. The coefficient for ZBI scores did not change significantly between these 2 models (OR, 1.02; 95% CI, 0.97-1.08) and was very similar to the model containing all 1409 dyads (OR, 1.03; 95% CI, 1.00-1.05). However, the *C* statistic for the model containing ZBI data collected before patients returned to the ED (*C*, 0.68; SE, 0.03) was greater than for the model including patients who returned to the ED before the ZBI data were collected (*C*, 0.61, SE, 0.03). The ability to correctly distinguish between patients who did and did not revisit within 30 days was enhanced when ZBI data were collected before the revisit (eAppendix 5 in Supplement 1).

## Discussion

We analyzed the association of caregiver burden of care with ED revisits and ED revisits with subsequent hospitalizations within 30 days of an index ED visit among a large cohort of community-dwelling older adults. We adjusted for factors associated with both caregiver burden and the tendency for repeat ED use. Caregiver ZBI scores were significantly associated with patient revisits at 30 days, holding important covariates constant. Other authors reported that caregiver burden, when treated as a continuous variable, was associated with ED use among patients with major neurocognitive disorders, but its effect size was small, as was the case in this study.^44^ The association of ZBI scores and precedent past-year ED visits with 30-day ED revisits was moderated by the COVID-19 pandemic waves, with the first interwave period attenuating the association, likely because older adults were encouraged to distance themselves from health care services.^45,46^ The models for revisits within 72 hours and within 7 days identified patient sex, patient and caregiver living conditions, CTAS score, and prior ED visits as factors associated with the likelihood of a revisit, but ZBI scores were not a significant factor in these shorter-term revisit models. An increased likelihood of 30-day hospitalization following an ED visit was associated with arriving as a walk-in, having a higher age-adjusted CCI score, and any reported caregiver income (whether high or low) compared with unreported income. We interpret these findings as evidence that caregiver burden may contribute to a negative care transition, associated with 30-day ED revisits, whereas shorter revisit intervals and hospitalizations may be more closely related to preexisting reliance on the ED and patient comorbidities.

The modest area under the receiver operating characteristic curve (*C* statistic) for all regression models is likely optimistic, considering that we did not fit the models to new data. Nonetheless, we suggest there are important missing variables at play, which might include the chronicity or acuteness of the reason for visiting the ED (eg, chronic heart failure vs myocardial infarction), the frailty of patients and caregivers, and the health professional seen in the ED prior to discharge (eg, consulting with a specialized geriatric emergency medicine nurse or a geriatrician). The modest *C* statistics also suggest a heterogenous sample of patients visiting the ED, but as the ED is an entrance to the health system, some heterogeneity in sampling is expected. Caregiver burden is also known to be a highly personal and intersectional experience, which poses additional challenges to heterogeneity.^47^

In most cases, we were able to collect the ZBI score before patients revisited the ED. While this presents a temporal bias, there is evidence to suggest that caregiver burden is stable at 5 months,^48^ 6 to 12 months,^49^ and 1 to 2 years^50^ after initial measurement. A previous study found that for caregivers in this study’s cohort already experiencing some burden, caregiver burden increased slightly following the index visit of an ED care transition.^51^ Further research on caregiver burden as a predictor of ED returns would benefit from a longitudinal design to assess burden levels at ED discharge and during follow-up to test if fluctuations in burden are short-term or indicative of long-term trends among caregivers. In practice at the ED, the addition of a long-form caregiver burden questionnaire may not be feasible, but there is an ultrashort single-item instrument (the ZBI-1) that could be tested prospectively to verify if systematic screening of caregiver burden in the ED and mitigation strategies put in place prior to ED discharge could reduce ED revisits.^28,52^ Some such strategies include involving caregivers as active members of the care team^53^ and assigning caregiver navigators who review and disseminate information about local support services throughout the care transition.^54^ Caregiver burden itself can be lessened through improving caregiving self-efficacy, with training on providing complex care,^55^ physician training on caregiver-centered care,^56^ and social support services designed to assist with chores and errands.^51,57,58^

### Strengths and Limitations

Strengths of our study include the inclusion of a large prospective cohort from both urban and rural communities, the use of psychometrically validated tools, and thorough regression fit testing and variable selection aimed at parsimony and alignment with theoretical frameworks. Limitations include potential selection biases. We administered our questionnaires via telephone calls, which may have prevented the participation of patients who hear less well, and we excluded patients with neurocognitive disorders, as was required by the ethics committee to ensure informed and competent consent, although caregivers of patients with neurocognitive disorders are known to have higher degrees of caregiver burden.^59,60^ Bias in responding may have arisen from patients and caregivers who may have responded to questionnaires in a socially desirable way. Most caregiver data were collected before the patient revisited the ED, and although ZBI score collection conducted before the revisit slightly changed the association between caregiver burden and 30-day ED revisits, it did not significantly alter model coefficients.

## Conclusions

In this cohort study of community-dwelling older adult patients and their caregivers, caregiver burden was associated with an increased likelihood of patient ED revisits within 30 days of the index visit, although not for shorter 3- and 7-day revisit intervals and not for revisits resulting in hospitalization. Our models had only modest performance, indicating potential missing variables and unaccounted heterogeneity in the study population. Future studies may consider measuring caregiver burden at patients’ ED discharge and leveraging longitudinal designs to deepen understanding of caregiver burden in relation to ED use. This may improve understanding of caregiver burden and its management and help to prevent ED revisits in older community-dwelling adults.

## Notes

Supplement 1. **eAppendix 1.** List of Variables Collected and Their Transformations **eAppendix 2.** A Priori Power Analysis **eAppendix 3.** Purposeful Selection Procedure as Outlined in Hosmer et al^40^ **eAppendix 4.** Model Output for 7-Day Revisits, 3-Day Revisits, and 30-Day Revisits Resulting in an Admission **eAppendix 5.** Model Output for Sensitivity Analyses

Supplement 2.
Nonauthor Collaborators

Supplement 3.
Data Sharing Statement
