# Supplementary material for: Caregiver Burden and 30-Day Emergency Department Revisits
Source: JAMA Netw Open. 2025 Sep 9;8(9):e2531166. doi: 10.1001/jamanetworkopen.2025.31166 (PMC12421348; doi:10.1001/jamanetworkopen.2025.31166)
Supplement: Supplement 1. — eAppendix 1. List of Variables Collected and Their Transformations eAppendix 2. A Priori Power Analysis eAppendix 3. Purposeful Selection Procedure as Outlined in Hosmer et al40 eAppendix 4. Model Output for 7-Day Revisits, 3-Day Revisits, and 30-Day Revisits Resulting in an Admission eAppendix 5. Model Output for Sensitivity Analyses [file jamanetwopen-e2531166-s001.pdf]

Supplementary Online Content

Germain N, Toulouse-Fournier A, Samb R, et al; LEARNING WISDOM investigators for the Network of Canadian Emergency Researchers. Caregiver burden and 30-day emergency department revisits. *JAMA Netw Open*. 2025;8(9):e2531166. doi:10.1001/jamanetworkopen.2025.31166

eAppendix 1. List of Variables Collected and Their Transformations

eAppendix 2. A Priori Power Analysis

eAppendix 3. Purposeful Selection Procedure as Outlined in Hosmer et al, 2013[40]

eAppendix 4. Model Output for 7-Day Revisits, 3-Day Revisits, and 30-Day Revisits Resulting in an Admission

eAppendix 5. Model Output for Sensitivity Analyses

This supplementary material has been provided by the authors to give readers additional information about their work.

© 2025 Germain N et al. *JAMA Network Open*.

eAppendix 1. List of variables collected and their transformations

Categorical or factor variables		
**Re-coded categorical variable**	**Re-coded levels (Translated)**	**Original levels as described in the French questionnaire**
Patient education level	Primary school*	Études primaires
	Secondary school	Études secondaires (DES)<br>Formation professionnelle (DEP, ASP)<br>Études collégiales (DEC)
	University	Baccalauréat<br>Études de cycles supérieurs (2e ou 3e cycle)
Caregiver education level	Primary school*	Études primaires
	Secondary school	Études secondaires (DES)<br>Formation professionnelle (DEP, ASP)<br>Études collégiales (DEC)
	University	Baccalauréat<br>Études de cycles supérieurs (2e ou 3e cycle)

© 2025 Germain N et al. *JAMA Network Open*.

Caregiver-Patient relation	Other family member or friend*	Soeur/frère<br>Amie/Ami<br>Petite-fille/petit-fils<br>Nièce/neveu<br>Belle-filles/beau-fils<br>Autre
	Spouse	Conjoint/Conjointe
	Parent-Child	Fils/Fille
Patient annual income ($ CAD)	< 30,000$	Moins de 10 000$<br>10 000 à 19 999$<br>20 000 à 29 999$
	> or equal to 30,000$	30 000 à 39 999$<br>40 000 à 49 999$<br>50 000 à 59 999$<br>60 000 à 69 999$<br>70 000 à 79 999$<br>80 000 à 89 999$<br>90 000 à 99 999$<br>Plus de 100 000$
	No response*	Préfère ne pas répondre

© 2025 Germain N et al. *JAMA Network Open*.

		Inconnu / manquant
Caregiver annual income ($ CAD)	< 50,000$	Moins de 10 000$<br>10 000 à 19 999$<br>20 000 à 29 999$<br>30 000 à 39 999$<br>40 000 à 49 999$
	> or equal to 50,000$	50 000 à 59 999$<br>60 000 à 69 999$<br>70 000 à 79 999$<br>80 000 à 89 999$<br>90 000 à 99 999$<br>Plus de 100 000$
	No response*	Préfère ne pas répondre<br>Inconnu / manquant
Patient residence type	Care home	Résidence privée pour personnes aînés avec présence infirmière 24h/24<br>Résidence privée pour personnes aînés sans infirmière sur place<br>Centre hospitalier de soins longue durée (CHSLD)

© 2025 Germain N et al. *JAMA Network Open*.

	Home, with others*	Domicile, partagé
	Home, alone	Domicile, seul<br><br>Ressources intermédiaires ou de type familial (RI ou RTF)<br><br>Habitation à loyer modique (HLM)
Caregiver residence type	Care home	Résidence privée pour personnes aînés avec présence infirmière 24h/24<br><br>Résidence privée pour personnes aînés sans infirmière sur place<br><br>Centre hospitalier de soins longue durée (CHSLD)
	Home, with others*	Domicile, partagé
	Home, alone	Domicile, seul<br><br>Ressources intermédiaires ou de type familial (RI ou RTF)<br><br>Habitation à loyer modique (HLM)

* Reference level

Numeric variables

**Variable name**	**Definition**	**Calculation**
**ZBI score**	Score of ZBI questionnaire	Sum of all ZBI items

© 2025 Germain N et al. *JAMA Network Open*.

**Time on stretcher (hours)**	Time patient spent on stretcher at the ED	Calculated by MedGPS
**Triage delay (hours)**	Delay between arrival at the ED and triage	Calculated by MedGPS
**Visits to the ED in the last year**	Number of unique visits to the ED 365 days before the index visit	Count of visits between 365 days before index and the index visit
**Visits to the ED in following 30 days**	Number of unique visits to the ED 30 days after the index visit	Count of visits between the index and the 30 days after the index visit
**Charlson Comorbidity Index**	Predicts the ten-year mortality for a patient with comorbid conditions	Weighted sum of comorbidity item scores
**Charlson Comorbidity Index (without age)**	Indice of comorbidity burden in each patient	Weighted sum of comorbidity item scores with the age-item removed
**Revisit at 30 days**	Whether or not a 30-day revisit occurred	Identifies whether a revisit occurred in the time between the date and time the patient left the ED + 30 days
**Revisit at 7 days**	Whether or not a 7-day revisit occurred	Identifies whether a revisit occurred in the time between the date and time the patient left the ED + 7 days

© 2025 Germain N et al. *JAMA Network Open*.

**Revisit at 3 days**	Whether or not a 3-day revisit occurred	Identifies whether a revisit occurred in the time between the date and time the patient left the ED + 3 days
**Revisit resulting in admission at 30 days**	Whether or not a 30-day revisit occurred resulting in admission	Identifies whether a revisit occurred in the time between the date and time the patient left the ED + 30 days AND the revisit was classified as an admission

© 2025 Germain N et al. *JAMA Network Open*.

eAppendix 2. A priori power analysis

We performed a-priori analyses to determine the estimated power to detect effects of interest. First, we assumed a very small effect size (OR = 1.2) of a positive association between ZBI scores and the likelihood of returning to the ED within 30 days, with a statistical significance level of 0.05. We also assumed a prevalence of 15% for 30-day ED visits. These estimates were based on previous work using the ZBI to predict hospital readmissions [1] and previous studies on ED use and revisit rates in older adult populations [2–6]. Using methods described by Demidenko [7] and Zhang and Yuan [8], power curves were plotted using logistic regression simulations with an ED revisit as the dependent variable, and the ZBI score as the single predictor variable. In simulations using normally distributed and lognormal ZBI scores [9,10], 1100 and 700 patients were sufficient to achieve a statistical power of 80%, respectively (Figures A and B).

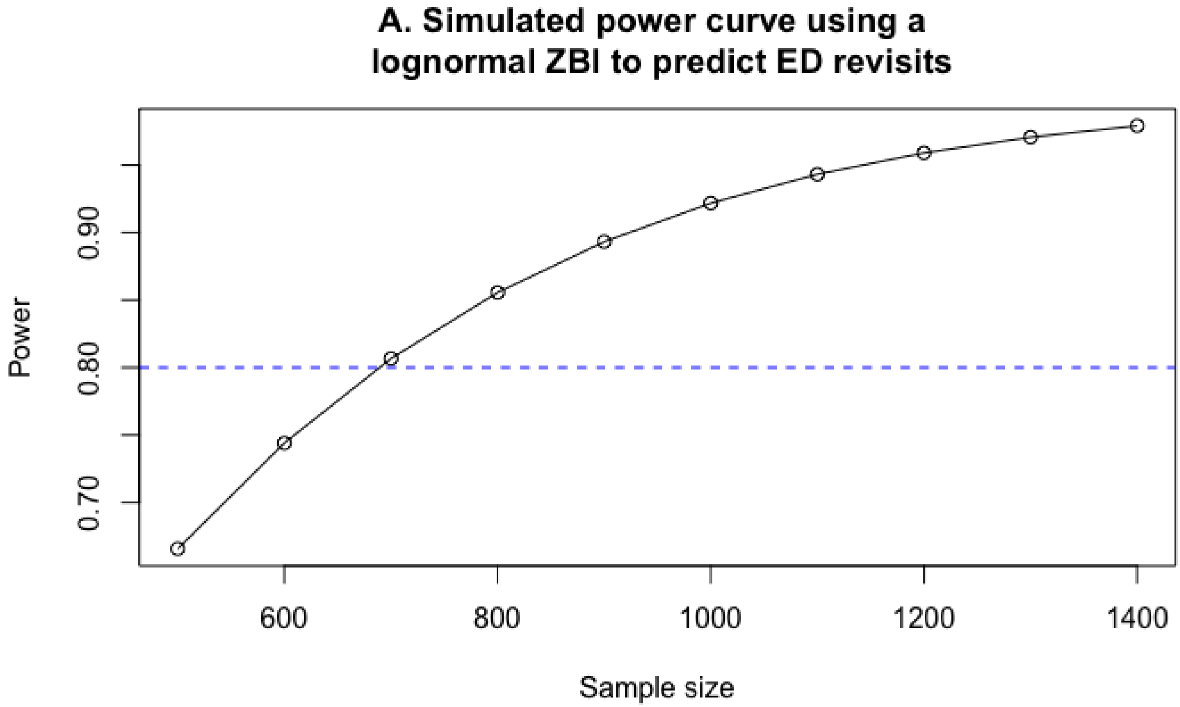

Note: assuming a lognormal distributed ZBI, and a revisit prevalence between 13 to 15%

© 2025 Germain N et al. *JAMA Network Open*.

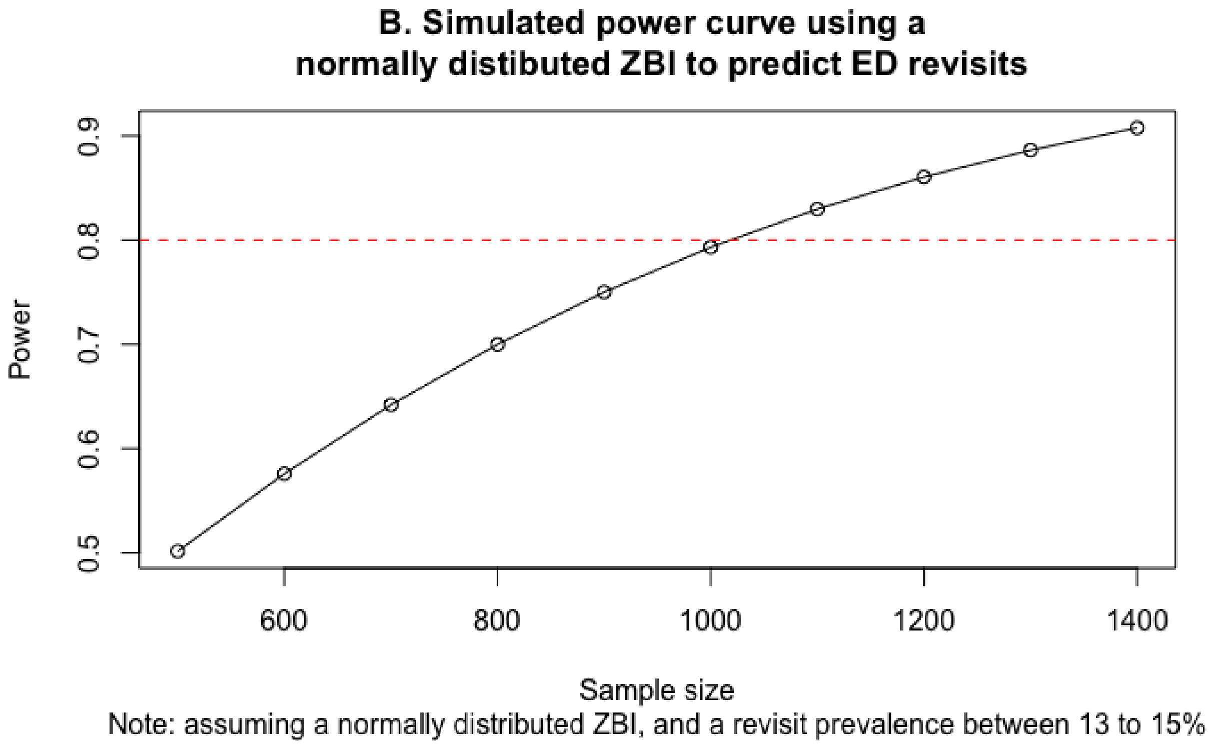

Simulations were then performed with the same parameters as described above to determine the number of covariates that could be accommodated while maintaining a statistical power of 80%. We had projected the number of caregiver-patient dyads to be between 1400 and 1500. For each successive logistic regression model assuming a normally distributed ZBI score, we added a covariate and an interaction effect between the ZBI scores and the covariate. Based on the results of these simulations, the model could accommodate a maximum of 3 covariates and 3 interaction terms with ZBI scores as the predictor variable (Figure C).

© 2025 Germain N et al. *JAMA Network Open*.

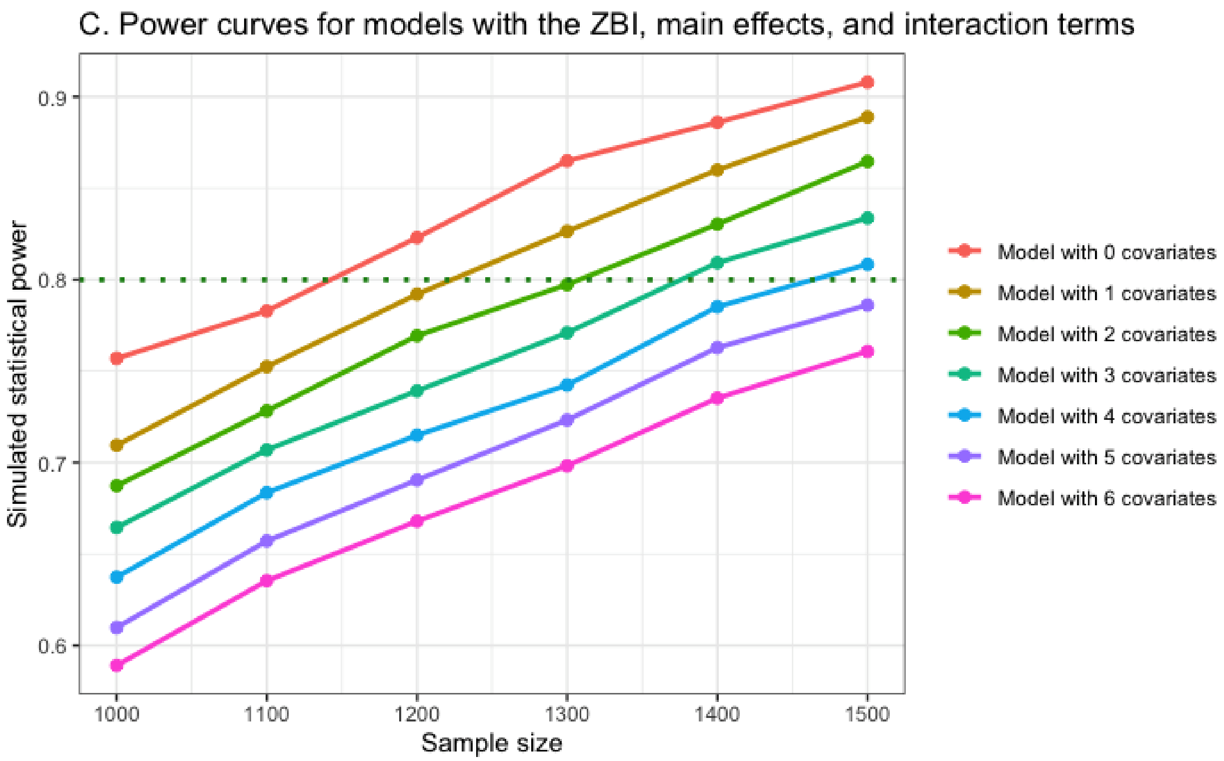

References for eAppendix 2

1. Fitriana I, Setiati S, Rizal EW *et al.* Malnutrition and depression as predictors for 30-day unplanned readmission in older patient: a prospective cohort study to develop 7-point scoring system. *BMC Geriatrics* 2021;**21**:256.

2. Gruneir A, Fung K, Fischer HD *et al.* Care setting and 30-day hospital readmissions among older adults: a population-based cohort study. *CMAJ* 2018;**190**:E1124–33.

3. Gruneir A, Cigsar C, Wang X *et al.* Repeat emergency department visits by nursing home residents: a cohort study using health administrative data. *BMC Geriatrics* 2018;**18**:157.

4. Simpson M, Sergi C, Malsch A *et al.* Association of Geriatric Emergency Department post-discharge referral order and follow-up with healthcare utilization. *J Am Geriatr Soc* 2023;**71**:821–31.

5. Hamilton MP, Bellolio F, Jeffery MM *et al.* Risk of falls is associated with 30-day mortality among older adults in the emergency department. *The American Journal of Emergency Medicine* 2024;**79**:122–6.

6. Sun BC, Burstin HR, Brennan TA. Predictors and Outcomes of Frequent Emergency Department Users. *Academic Emergency Medicine* 2003;**10**:320–8.

7. Demidenko E. Sample size determination for logistic regression revisited. *Statistics in Medicine* 2007;**26**:3385–97.

© 2025 Germain N et al. *JAMA Network Open*.

8. Zhang Z, Yuan K-H. *Practical Statistical Power Analysis Using Webpower and R*., 2018.

9. Flynn Longmire CV, Knight BG. Confirmatory factor analysis of a brief version of the Zarit Burden Interview in Black and White dementia caregivers. *Gerontologist* 2011;**51**:453–62.

10. Hagell P, Alvariza A, Westergren A *et al.* Assessment of Burden Among Family Caregivers of People With Parkinson's Disease Using the Zarit Burden Interview. *J Pain Symptom Manage* 2017;**53**:272–8.

© 2025 Germain N et al. *JAMA Network Open*.

eAppendix 3. Purposeful selection procedure as outlined in Hosmer et al., 2013.

Step 1 of this method involved conducting univariate tests of covariates on the outcome variable, selecting any variables with a *p-value* < .25. *Step 2* used all the covariates identified in *Step 1* to construct a base model. In *Step 3*, iterative variable selection was conducted, and non-significant covariates (*p-value* < .05) were removed unless they acted as confounders, identified by changes exceeding 20% in parameter estimates. In *Step 4*, any variables not selected in *Step 2* were added back into the model. If any of these variables showed statistically significant main effects, they were added back into the model, which was then our *preliminary main effects model*. In *Step 5*, the assumption of linearity was tested for each continuous variable. This assumption of linearity in the logit referred to the linear relation between the log odds of the outcome variable and the predictor variables. In *Step 6*, the main effects in the model were tested for interaction effects. Interaction between two covariates implied that the effect of each variable was not constant over levels of the other variable. Lastly, in *Step 7*, goodness of fit was assessed, along with an evaluation of the discriminatory power of this final model using a receiver operating characteristic (ROC) curve.

© 2025 Germain N et al. *JAMA Network Open*.

eAppendix 4. Model output for 7-day revisits, 3-day revisits and 30-day revisits resulting in an admission.

A. Model characteristics from logistic regression model on 7-day revisits

**Characteristic**	**Univariate OR[1]**	**Multivariate OR[1]**	**95% CI[1]**	***p*-value**
**ZBI Score**	1.01	1.01	0.98, 1.03	0.6
**Previous ED visits**	1.14	1.15	1.05, 1.25	**0.002**
**Time on stretcher at the ED**	0.98	0.98	0.96, 1.00	**0.059**
**Patient sex**				
Man	—	—	—	
Woman	0.60	0.60	0.41, 0.88	**0.010**
**Patient residence type**				
Home	—	—	—	
Home, alone	1.16	1.55	0.95, 2.52	0.077
Care home	0.68	0.63	0.33, 1.20	0.160
**Caregiver residence type**				
Home	—	—	—	
Home, alone	0.81	0.71	0.39, 1.30	0.271
Care home	3.21	3.84	1.32, 11.15	**0.013**
**Triage (CTAS) on index visit**				
5	—	—	—	
4	1.81	1.95	0.97, 3.90	**0.060**
3	1.89	1.96	0.96, 4.00	**0.064**
2	3.51	3.95	1.54, 10.18	**0.004**

© 2025 Germain N et al. *JAMA Network Open*.

Characteristic	Univariate OR[1]	Multivariate OR[1]	95% CI[1]	*p*-value

[1]OR = Odds Ratio, CI = Confidence interval for multivariate OR

© 2025 Germain N et al. *JAMA Network Open*.

B. Model characteristics from logistic regression model on 3-day revisits

Characteristic	Univariate OR[1]	Multivariate OR[1]	95% CI[1]	*p*-value
**ZBI Score**	1.01	1.01	0.98, 1.04	0.69
**Previous ED visits**	1.13	1.13	1.01, 1.25	**0.023**
**Caregiver residence type**				
Home	—	—	—	
Home, alone	1.01	0.94	0.47, 1.88	0.86
Care home	3.70	3.37	1.10, 10.33	**0.033**
**Triage (CTAS) on index visit**				
5	—	—	—	
4	3.37	3.59	1.09, 11.86	**0.036**
3	3.85	3.78	1.13, 12.61	**0.030**
2	6.75	7.14	1.71, 29.72	**0.007**
**Time on stretcher at the ED**	0.97	0.97	0.94, 1.00	**0.035**

[1] OR = Odds Ratio, CI = Confidence interval for multivariate OR

© 2025 Germain N et al. *JAMA Network Open*.

C. Model characteristics from logistic regression model on 30-day revisits resulting in admission

**Characteristic**	**Univariate OR**[1]	**Multivariate OR**[1]	**95% CI**[1]	**p-value**
**ZBI Score**	1.02	1.02	0.99, 1.05	0.24
**Charlson Score**	1.18	1.17	1.07, 1.29	**<0.001**
**Arrival method**				
Ambulance	—	—	—	
Ambulant	1.46	1.57	1.01, 2.43	**0.046**
**Caregiver annual income**				
No response	—	—	—	
< 50,000$	2.73	2.81	1.47, 5.38	**0.002**
> or equal to 50,000$	2.02	2.12	1.05, 4.26	**0.035**

[1]OR = Odds Ratio, CI = Confidence interval for multivariate OR

© 2025 Germain N et al. *JAMA Network Open*.

eAppendix 5. Model output for sensitivity analyses

	ZBI collected before revisit			ZBI collected after revisit		
**Characteristic**	**OR[1]**	**95% CI[1]**	**p-value**	**OR[1]**	**95% CI[1]**	**p-value**
**ZBI Score**	1.02	0.96, 1.07	0.4	1.02	0.97, 1.08	0.4
**Previous ED visits**	1.03	0.77, 1.29	0.8	1.05	0.89, 1.30	0.6
**Covid-19 Period**						
Pre-pandemic	—	—		—	—	
Wave 1	0.80	0.19, 2.84	0.7	0.43	0.09, 2.00	0.3
Between Wave 1 and Wave 2	3.93	0.79, 17.3	**0.075**	1.45	0.24, 13.3	0.7
Wave 2	1.13	0.35, 3.45	0.8	1.59	0.49, 5.45	0.4
Wave 3	0.20	0.01, 1.77	0.2	0.39	0.01, 7.14	0.5
Wave 4	1.72	0.26, 8.32	0.5	1.29	0.25, 7.70	0.8
**ZBI Score * Covid-19 Period**						

© 2025 Germain N et al. *JAMA Network Open*.

	ZBI collected before revisit			ZBI collected after revisit		
**Characteristic**	**OR[1]**	**95% CI[1]**	**p-value**	**OR[1]**	**95% CI[1]**	**p-value**
*ZBI Score * Wave 1*	1.09	0.99, 1.21	**0.074**	1.02	0.91, 1.16	0.7
*ZBI Score * Between Wave 1 and Wave 2*	0.82	0.59, 1.00	0.13	0.96	0.78, 1.22	0.7
*ZBI Score * Wave 2*	1.01	0.91, 1.10	>0.9	1.00	0.90, 1.12	>0.9
*ZBI Score * Wave 3*	0.98	0.80, 1.15	0.9	1.24	0.94, 2.17	0.3
*ZBI Score * Wave 4*	0.91	0.68, 1.07	0.4	1.03	0.87, 1.26	0.7
**Previous ED visits * Covid-19 Period**						
*Previous ED visits * Wave 1*	0.81	0.38, 1.42	0.5	1.44	0.91, 2.55	0.2
*Previous ED visits * Between Wave 1 and Wave 2*	1.44	0.70, 2.92	0.3	0.94	0.52, 1.88	0.8
*Previous ED visits * Wave 2*	1.03	0.67, 1.53	0.9	0.96	0.67, 1.43	0.8
*Previous ED visits * Wave 3*	2.16	1.22, 4.85	**0.029**	1.03	0.46, 2.48	>0.9

© 2025 Germain N et al. *JAMA Network Open*.

	ZBI collected before revisit			ZBI collected after revisit		
**Characteristic**	**OR[1]**	**95% CI[1]**	**p-value**	**OR[1]**	**95% CI[1]**	**p-value**
*Previous ED visits * Wave 4*	1.16	0.69, 1.81	0.5	0.79	0.49, 1.24	0.3

[1] OR = Odds Ratio, CI = Confidence Interval

© 2025 Germain N et al. *JAMA Network Open*.
