# Supplementary material for: Caregiver Burden and 30-Day Emergency Department Revisits
Source: JAMA Netw Open. 2025 Sep 9;8(9):e2531166. doi: 10.1001/jamanetworkopen.2025.31166 (PMC12421348; doi:10.1001/jamanetworkopen.2025.31166)
Supplement: Supplement 2. — Nonauthor Collaborators [file jamanetwopen-e2531166-s002.pdf]

Supplemental Online Content: Nonauthor Collaborators
*First name, last name, and suffix (if applicable) are required and will appear in PubMed.

*Group Name(s): LEARNING WISDOM investigators; for the Network of Canadian Emergency Researchers									
***First Name and Middle Initial(s)**	***Last Name**	***Suffix (eg, Jr, III)**	Academic Degrees	Institution	Location (city, state/province, country)	Role or Contribution, eg, chair, principal investigator	Group (if more than 1 Group listed in the byline) and/or Subgroup (eg, Steering Committee)		
Sam	Chandavong		BSc	Centre de recherche intégrée pour ur	Lévis, Québec, Canada	Programmer	LEARNING WISDOM investigators		
Raphaëlle	Giguère		MSc	Centre de recherche intégrée pour ur	Lévis, Québec, Canada	Programmer	LEARNING WISDOM investigators		
Lyna	Abrougui		BSc	Centre de recherche intégrée pour ur	Lévis, Québec, Canada	Programmer	LEARNING WISDOM investigators		
Clémence	Dallaire		PhD	Centre de recherche intégrée pour ur	Lévis, Québec, Canada	Co-Investigator	LEARNING WISDOM investigators		
Lise	Lavoie		BSc	Centre de recherche intégrée pour ur	Lévis, Québec, Canada	Administrative	LEARNING WISDOM investigators		
Laetitia	Bert		MA	Centre de recherche intégrée pour ur	Lévis, Québec, Canada	Collaborator	LEARNING WISDOM investigators		
Marcel	Émond		MD, MSc	Faculty of Medicine, Université Laval	Québec, Québec, Canada	Co-Investigator	Network Of Canadian Emergency Researchers		
Annie	LeBlanc		PhD	Faculty of Medicine, Université Laval	Québec, Québec, Canada	Co-Investigator	LEARNING WISDOM investigators		
Audrey-Anne	Brousseau		MD	CIUSSSE - Centre Hospitalier Universi	Sherbrooke, QC, Canada	Co-Investigator	Network Of Canadian Emergency Researchers		
Isabelle	Pelletier		BSc	Centre de recherche intégrée pour ur	Lévis, Québec, Canada	Administrative	LEARNING WISDOM investigators		
Jean-Louis	Denis		PhD	Institute of Health Policy, Manageme	Toronto, ON, Canada	Co-Investigator	LEARNING WISDOM investigators		
