# Supplementary material for: Caregiver Burden and 30-Day Emergency Department Revisits
Source: JAMA Netw Open. 2025 Sep 9;8(9):e2531166. doi: 10.1001/jamanetworkopen.2025.31166 (PMC12421348; doi:10.1001/jamanetworkopen.2025.31166)
Supplement: Supplement 3. — Data Sharing Statement [file jamanetwopen-e2531166-s003.pdf]

Data Sharing Statement

Germain. Caregiver Burden and 30-Day Emergency Department Revisits. *JAMA Netw Open*. Published September 09, 2025. doi:10.1001/jamanetworkopen.2025.31166

Data
Data available: No

Additional Information
Explanation for why data not available: Analysis code is consultable in a public GitHub repository (https://github.com/LMD-nat/fardeau). A data dictionary is provided in the supplement. Anonymized data are available from the corresponding author on reasonable request.
